# An atypical presentation of Kikuchi‐Fujimoto disease: A case report & literature review

**DOI:** 10.1002/ccr3.3373

**Published:** 2020-11-04

**Authors:** Abdul Rahman R. Halawa, Muayad K. Ahmad, Abdulqadir J. Nashwan

**Affiliations:** ^1^ Medical Education Hamad Medical Corporation (HMC) Doha Qatar; ^2^ Department of Medicine Hazm Mebaireek General Hospital (HMGH) Hamad Medical Corporation (HMC) Doha Qatar; ^3^ Weill Cornell Medical College in Qatar (WCM‐Q) Doha Qatar; ^4^ Hazm Mebaireek General Hospital (HMGH) Hamad Medical Corporation (HMC) Doha Qatar; ^5^ University of Calgary in Qatar (UCQ) Doha Qatar

**Keywords:** autoimmune hemolytic anemia, histiocytic necrotizing lymphadenitis, Kikuchi‐Fujimoto disease, systemic lupus erythematosus

## Abstract

The authors urge clinicians to consider the possibility of Kikuchi‐Fujimoto Disease associated with autoimmune hemolytic anemia with possible correlation with systemic lupus erythematosus in patients presented with lymphadenopathy and fever.

## Graphical abstract

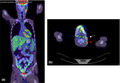

## 1 BACKGROUND

Kikuchi‐Fujimoto disease (KFD) is a self‐limiting condition that typically presents with lymphadenopathy and fever. It is autoimmune in nature and possibly associated with systemic lupus erythematosus (SLE). The case report highlights an unusual case of KFD associated with autoimmune hemolytic anemia and provided a review for other atypical presentations.

Kikuchi Disease, also known as Kikuchi‐Fujimoto disease (KFD), is a benign, self‐limiting condition of undetermined etiology, that commonly presents with lymphadenopathy and fever. The disease was initially described in young women from Japan. However, it became more apparent that although Kikuchi is frequently reported in Asia, it does occur in various racial groups. In a retrospective literature review by Kucukardali et al,^1^ cases were reported from the USA, Taiwan, and Spain, with a male to female ratio of 1:3.

Although the pathogenesis is not completely understood, the underlying mechanism is thought to be an immune response to an infectious agent, with some studies reporting Epstein‐Barr virus (EBV),^2^ Human herpesvirus 6 (HHV6),^3^ parvovirus,^4^ and others reporting bacterial and parasitic infection. The immune response predominantly involves T cells and histiocytes, in addition to elevated levels of cytokines, markedly interferon‐gamma and IL‐6.^5^

The most common presentation in the majority of cases of KFD is cervical lymphadenopathy. In a literature review by Kucukardali et al,^1^ out of 244 cases of KFD, 100% had cervical lymphadenopathy, and 35% of cases complained of fever. Other reported signs and symptoms include rash, arthritis, and hepatosplenomegaly.

Although anemia associated with mild microcytosis is an observed laboratory finding in patients with KFD^6^ accompanying autoimmune hemolytic anemia is unusual. Other atypical presentations features may include pan‐uveitis,^7^ thyroiditis,^8^ cerebellar ataxia,^9^ autoimmune hepatitis,^10^ and peripheral neuropathy.^11^

The histological features of KFD can mimic that of SLE, Lymphoma, and tuberculous adenitis, in addition, herpes simplex lymphadenitis.^12^

However, these can be differentiated by distinct microscopic and immuno‐architectural features. For example, in the case of lymphoma and reactive lymphadenitis, Kishomoto et al suggested that plasmacytoid dendritic cells are more abundant in the paracortical infiltrate in cases of KFD.^13^ Additionally, CD8^+^ T cells are more prominent around areas of necrosis in KFD, and unlike SLE.^14^

In another case series by Dorfman et al^15^, fever was a predominant symptom, seen in up to 50% of the cases. Diagnosis is made by lymph node biopsy, which typically has 2 phases, the early proliferative phase and the necrotizing phase. Xanthomatous changes can also be seen in some reported cases, but this is believed to be a separate variant.^16^

Kikuchi‐Fujimoto disease is usually self‐remitting, and the resolution of symptoms is usually seen within 6 months of presentation. However, some reports describe incidents of disease recurrence and the development of morbidities. In a case series by Song et al, 8 out of 102 patients with KFD developed early relapse, and 13 demonstrated late recurrence. Additionally, 3 developed Systemic lupus erythematosus (SLE). Additionally, they reported an association between the recurrence rate and positive fluorescence anti‐nuclear antibody (FANA) testing.^17^

Although anemia associated with mild microcytosis is an observed laboratory finding in patients with KFD^18^ accompanying autoimmune hemolytic anemia is unusual. In this article, we present a case of KFD with accompanying autoimmune hemolytic anemia, and we review the literature of possible etiologies and associations of the disease, in addition to other atypical presentations.

## 2 CASE PRESENTATION

We describe a 28‐year‐old Bangladeshi male, with no significant past medical history, that was referred to our institution with a 4‐day history of fever, and a 1‐month history of generalized fatigue and 6 kg weight loss. The patient denied night sweats, cough, dyspnea, shortness of breath, chest pain, headache, dysuria, diarrhea, vomiting, or constipation. His history was negative for a viral prodrome of including rhinorrhea or sore throat. He also denied sick contacts or recent travel. Examination revealed painless bilateral cervical lymphadenopathy, in addition to splenomegaly, jaundice, and pallor.

The patient was found to have severe anemia Hgb 5.9 (13‐17 g/dL), associated with an increased reticulocyte count Retic # 177.1 (50.0‐100.0 × 10^9^/L), Retic % 5.4 (0.5%‐2.5%), Bilirubin T 57.1 (3.4‐20.5 µmol/L), Bilirubin D 9.0 (0.0‐8.6 mg/dL), Lactic acid dehydrogenase (LDH) 546 (125‐220 U/L), direct antiglobulin positive and decreased haptoglobin <10 (30‐200 mg/dL). Additionally, laboratories revealed mild thrombocytopenia 108 (150‐400 × 10^3^/uL), markedly elevated ferritin 1222 (48‐420 ug/L), low iron, Total iron‐binding capacity (TIBC), and transferrin, as well as low B12. A peripheral blood smear revealed anisocytosis and poikilocytosis, target cells, oval, teardrop cells, spherocytes, basophilic stippling, Howell‐Jolly bodies, polychromasia, and red cells fragments with many Nucleated red blood cells (NRBCs) seen with dysplastic forms and was suggestive of hemolysis. Direct Coombs test was positive.

Computed tomography (CT) scan of the thorax and abdomen revealed centrilobular lung nodular opacities (likely infectious/inflammatory), bilateral hilar and mediastinal lymphadenopathy, in addition to prevascular, right paratracheal, and subcarinal lymphadenopathy, and marked osteopenia. Autoimmune hemolytic anemia secondary to lymphoma was suspected, and the patient underwent an excisional biopsy of a left cervical lymph node.

The patient was initially admitted at one of institutions hospitals, where he was admitted for a week, received multiple transfusions and underwent US‐guided needle biopsy and then excisional biopsy, his Hb was stable over for than 72 hours and was discharged for follow‐up of the excisional biopsy pathology report in the clinic. However, after 1 week the patient was seen in the clinic, laboratories revealed an Hb <7, he was admitted to the hospital, received transfusions, and his first excisional biopsy revealed Histiocytic Necrotizing Lymphadenitis. His Lymphadenopathy was still significant was the same overtime (not improving or worsening) on daily physical examinations. The suspicion for lymphoma was high so we did not give steroids (same reason as above) and underwent a second excisional biopsy which confirmed the diagnosis. He was admitted the second time for 2 weeks. After the 2nd excisional biopsy, he did not need blood transfusions and his Hb was >7 for more than 72 hours, steroids were not started and he was discharged. He was seen in the clinic again 1 week later, Hb was stable, still had lymphadenopathy that has not improved or worsened. Then, he was lost to follow‐up.

Pathology review of the sample at our institution revealed unusual patchy paracortical areas of atypical cells with increased apoptotic debris and histiocytes, in addition to lymphocytic infiltrates. The differential of which includes lymphoproliferative disorders vs reactive conditions such as the proliferative phase of KFD. The sample was sent to Mayo Clinic for expert evaluation. The lymphocytic infiltrates contained abundant CD2, CD3, CD5, and CD7 positive T cells, with CD8 positive T cells that outnumber CD4 positive cells in these areas. Histiocytes were positive for CD68, and a subset was positive for myeloperoxidase. Hodgkin cells were not identified in the stains for CD30 and CD50, and no Epstein‐Barr virus‐positive cells were identified. A diagnosis of Histiocytic Necrotizing Lymphadenitis was made. The immuno‐architectural features could represent KFD; however, identical morphological features can also be observed in cases of systemic lupus erythematosus.

**FIGURE 1:**
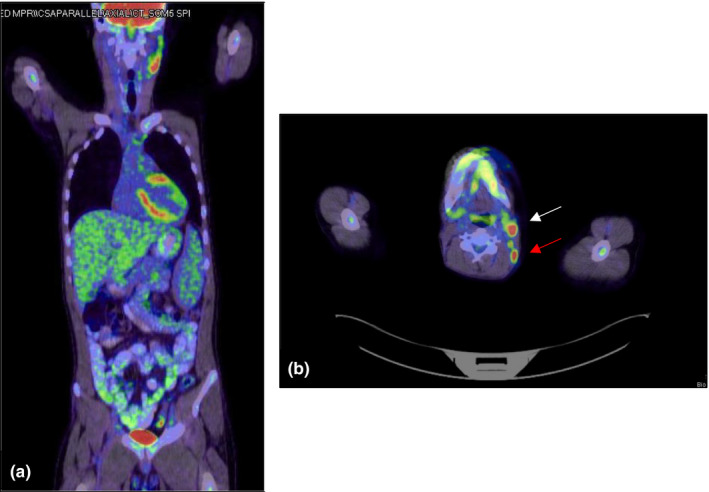
A, Hypermetabolic left cervical lymph node, 1.3 cm in diameter, SUV of 12.2. (White arrow). B, Left posterior neck is also showing few nodes around 7‐9 mm with SUV extending up to 7.7. (Red Arrow)

**FIGURE 2:**
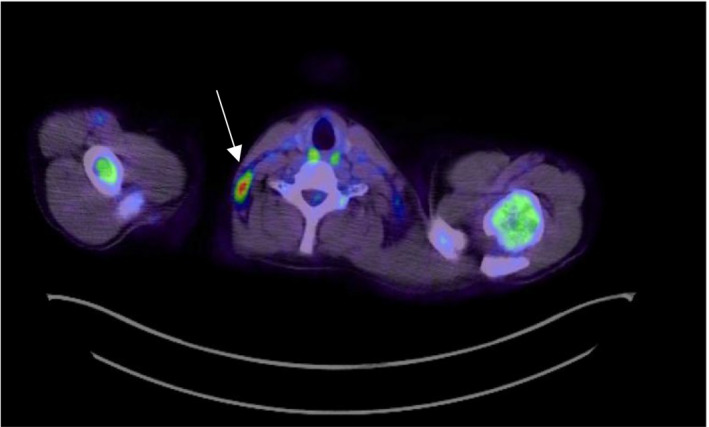
Hypermetabolic right cervical lymph node, 0.9 cm in size, SUV of 8.5. (White arrow)

**FIGURE 3:**
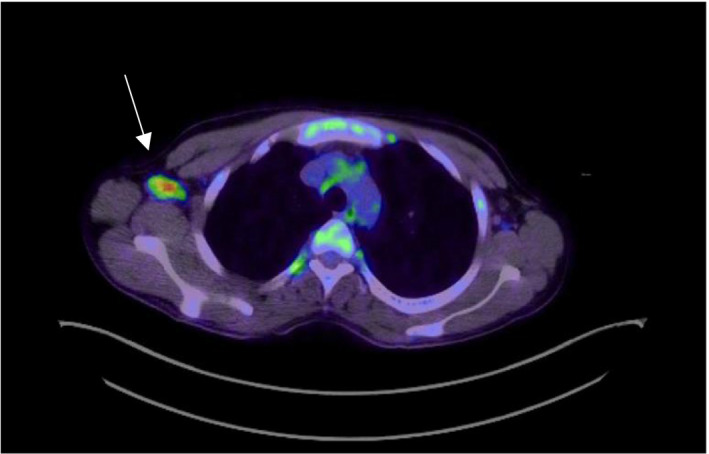
Hypermetabolic right axillary lymph node, 1 cm size, SUV up to 6.9. (White arrow)

Upon further investigation, the patient's ANA, antidouble‐stranded DNA, and anti‐Smith antibodies were negative. A Positron Emission Tomography—Computed Tomography (PET/CT) revealed multiple hypermetabolic nodes, included bilateral cervical nodes, the largest measuring 1.3 cm (SUV up to 12.2) located in the left upper neck (Figure 1A,B), and a node measuring 9 mm (SUV extending up to 8.5) in the right lower neck (Figure 2). Hypermetabolic nodes were also identified in the axilla (Figure 3), mediastinum, left hilum, and portocaval region, raising the suspicion for lymphoma, despite a negative excisional biopsy.

Bone marrow aspiration (BM) showed that the aspirate smears were cellular, partially hemodilution with increased erythroid precursors with some dysplastic changes, but no increase in blasts. The report stated: "The suboptimal BM biopsy reflects hypercellular marrow (almost 100% cellularity) showing trilineage hemopoiesis with increased erythroid precursors with focal areas showing increased in reticulin fiber and marrow vasculature. No lymphoid aggregate. No definite granuloma noted. The erythroid hyperplasia is secondary to the hemolytic anemia (hemoglobinopathy). Causes of increased fibrosis may include medication, immune disease, chronic disorders, chronic inflammatory diseases, and infection, among other causes."

The patient underwent a second excisional biopsy of the right cervical lymph node. As stated in the report, “sections show a lymph node in which the paracortical areas are expanded by a polymorphous lymphohistiocytic infiltrate with abundant karyorrhexis and apoptosis." The histiocytic cells are positive for CD68 and CD163, and a subset is positive for myeloperoxidase. TdT, c‐myc, ALK‐1, EBV LMP1, HHV8 are negative. A diagnosis of Histiocytic Necrotizing Lymphadenitis was made.

During his 4‐week hospital course, the patient did require multiple blood transfusions but did not receive steroids. Toward the end of his hospital course, his hemoglobin stabilized without transfusions or steroids. Upon follow‐up in the clinic 2 weeks after discharge, he reported overall improvement of his symptoms but continues to be jaundiced, and examination did reveal persistent cervical lymphadenopathy, in addition to clubbing, and splenomegaly. His hemoglobin was stable and is being monitored during his hematology follow‐up appointments.

He was jaundiced but his Hb remained stable, and because of the self‐remitting characteristics of the disease we decided to observe without intervention.

## 3 DISCUSSION

Autoimmune hemolytic anemia (AIHA) is relatively rare disorder caused by antibody destruction of erythrocytes. It can be idiopathic or secondary, and classified as warm, cold, or mixed. AIHA may develop gradually, or have a fulminant onset with life‐threatening anemia.^19^ The treatment of AIHA is still controversial. For warm AIHA, the first‐line therapy are corticosteroids. For refractory/relapsed cases, the second‐line therapy is splenectomy, rituximab, and immunosuppressive drugs (eg, azathioprine, cyclophosphamide, and cyclosporine). Additional therapies are intravenous immunoglobulins, danazol, plasma‐exchange, and alemtuzumab and high‐dose cyclophosphamide as last resort option.^20^

Autoimmune hemolytic anemia is a key presenting or historical feature of SLE and has been reported in about 10% of SLE patients.^21^ The presentation of AIHA in SLE is not unique compared to other conditions and includes anemia with an increased reticulocyte count, increased indirect bilirubin and lactate dehydrogenase (LDH), low serum haptoglobin, and a positive direct antiglobulin (Coombs) test; spherocytes should be seen on the peripheral blood smear which was typically presented in our patient.

Hodgkin's Lymphoma usually exhibits CD15, 30 and is negative for CD45. In this case, our patient's clinical picture was highly suggestive of lymphoma, and a second excisional lymph node biopsy was undertaken to confirm KFD and avoid misdiagnosis of lymphoma and subsequent cytotoxic treatment.

To our knowledge, no cases of KFD have been reported with associated autoimmune hemolytic anemia. Although most cases of AIHA are idiopathic, secondary AIHA can be due to autoimmune disease, including SLE. In a case series reported by Gormezano et al,^22^ 49 out of 1830 adults with SLE had diagnosed AIHA.

Multiple reports and case series underline a possible association between Kikuchi and SLE. Kucukardali et al's^1^ analysis of 244 cases of KFD, 32 had associated SLE. Dorfman et al report that 2 out of 108 cases of KFD developed SLE, and Singh et al reports 3 cases out of 102.^15^ Additionally, Baenas et al presented three patterns of SLE‐associated KFD; before the onset of SLE (20 cases), after SLE (11 cases), and simultaneous with SLE (14 cases). These cases were reported as early as 1991, up to the period of 2015.^23^

Kikuchi may be a self‐limited SLE‐like condition or mimic, which may be secondary to virally infected lymphocytes.^6^ Additionally, Kikuchi may be a clinical or histopathological phase of lupus lymphadenitis.^20^ On the contrary, many reported cases had no association with SLE.^1^

This introduces the question of whether patients with diagnosed KFD require periodical serological or clinical follow‐up for the development of SLE. Some authors do recommend follow up, especially for patients who develop severe complications^24^ or those with cutaneous manifestations.^25^, ^26^

Although the association is unclear, no guidelines have been set on the follow up of patients with Kikuchi, and no indications for rheumatology referral have been established.

## 4 CONCLUSION

Kikuchi‐Fujimoto Disease (KFD) is a benign, self‐limiting condition that typically presents with lymphadenopathy and fever, and resolves spontaneously. We report a case of KFD associated with autoimmune hemolytic anemia, which to our knowledge, has never been reported. The association between KFD and SLE has been reported in the literature, and our case is suggestive of an autoimmune etiology as the underlying etiology of KFD. Although the SLE workup for our patient was negative, the presence of autoimmune hemolytic anemia merits rheumatology referral and follow‐up for possible development of SLE. We also highlight the importance of considering KFD in patients who present with lymphadenopathy. Although our patient's presentation was highly suspicious of lymphoma, two excisional biopsies were undertaken to rule out lymphoma, and to avoid a misdiagnosis of lymphoma and subsequent cytotoxic treatment.

## CONFLICT OF INTEREST

The authors declare that they have no competing interests.

## AUTHOR CONTRIBUTIONS

ARH: involved in data collection, literature search, and manuscript preparation. MKA and AJN: involved in manuscript preparation. All authors read and approved the final manuscript.

## ETHICAL APPROVAL

The article describes a case report. Therefore, no additional permission from our Ethics Committee was required.

## CONSENT FOR PUBLICATION

The consent for publication was obtained.

## Data Availability

All data generated or analyzed during this study are included in this published article.
